# Vitamin D-responsive *SGPP2* variants associated with lung cell expression and lung function

**DOI:** 10.1186/1471-2350-14-122

**Published:** 2013-11-25

**Authors:** Brian J Reardon, Joyanna G Hansen, Ronald G Crystal, Denise K Houston, Stephen B Kritchevsky, Tamara Harris, Kurt Lohman, Yongmei Liu, George T O’Connor, Jemma B Wilk, Jason Mezey, Chuan Gao, Patricia A Cassano

**Affiliations:** 1 Division of Nutritional Sciences, Cornell University, 209 Savage Hall, Ithaca, NY 14853, USA; 2 Department of Genetic Medicine, Weill Cornell Medical College, New York, NY, USA; 3 Sticht Center on Aging, Wake Forest School of Medicine, Winston-Salem, NC 27157, USA; 4 Intramural Research Program, National Institute on Aging, Laboratory of Epidemiology, Demography, and Biometry, Gateway Building, 3C309, 7201 Wisconsin Avenue, Bethesda, MD 20892, USA; 5 Division of Public Health Sciences, Department of Biostatistical Sciences, Wake Forest School of Medicine, Winston-Salem, NC 27157, USA; 6 Division of Public Health Sciences, Department of Epidemiology & Prevention, Wake Forest School of Medicine, Winston-Salem, NC 27157, USA; 7 Section of Pulmonary, Allergy & Critical Care Medicine, Department of Medicine, Boston University School of Medicine, Boston, MA, USA; 8 The National Heart, Lung, and Blood Institute’s Framingham Heart Study, Framingham, MA, USA; 9 Division of Aging, Department of Medicine, Brigham and Women’s Hospital and Harvard Medical School, Boston, MA, USA; 10 Biological Statistics and Computational Biology, Cornell University, Ithaca, NY 14853, USA; 11 Department of Medical Genetics, Weill Cornell Medical College, New York, NY, USA; 12 Division of Biostatistics and Epidemiology, Department of Public Health, Weill Cornell Medical College, New York, NY, USA

**Keywords:** Vitamin D, Airflow obstruction, FEV_1_, *SGPP2*, FEV_1_/FVC

## Abstract

**Background:**

Vitamin D is associated with lung health in epidemiologic studies, but mechanisms mediating observed associations are poorly understood. This study explores mechanisms for an effect of vitamin D in lung through an *in vivo* gene expression study, an expression quantitative trait loci (eQTL) analysis in lung tissue, and a population-based cohort study of sequence variants.

**Methods:**

Microarray analysis investigated the association of gene expression in small airway epithelial cells with serum 25(OH)D in adult non-smokers. Sequence variants in candidate genes identified by the microarray were investigated in a lung tissue eQTL database, and also in relation to cross-sectional pulmonary function in the Health, Aging, and Body Composition (Health ABC) study, stratified by race, with replication in the Framingham Heart Study (FHS).

**Results:**

13 candidate genes had significant differences in expression by serum 25(OH)D (nominal p < 0.05), and a genome-wide significant eQTL association was detected for *SGPP2.* In Health ABC, *SGPP2* SNPs were associated with FEV_1_ in both European- and African-Americans, and the gene-level association was replicated in European-American FHS participants. SNPs in 5 additional candidate genes (*DAPK1, FSTL1, KAL1, KCNS3,* and *RSAD2*) were associated with FEV_1_ in Health ABC participants.

**Conclusions:**

*SGPP2,* a sphingosine-1-phosphate phosphatase, is a novel vitamin D-responsive gene associated with lung function. The identified associations will need to be followed up in further studies.

## Background

Vitamin D is of interest in relation to a number of health outcomes, with putative function beyond its classical role in maintaining bone health. The active form of vitamin D, 1,25-dihydroxyvitamin D [1,25(OH)_2_D], when bound to the vitamin D receptor (VDR), regulates the expression of genes in many molecular pathways, including inflammation, cell proliferation, cell death, and tissue-remodeling pathways [1]. Serum 25-hydroxyvitamin D [25(OH)D] is the primary circulating biomarker of vitamin D status, and recent national survey data in the U.S. indicate 32% of Americans are at risk of vitamin D inadequacy or deficiency, defined as 30–49 nmol/L and <30 nmol/L serum 25(OH)D, respectively [2,3].

Chronic obstructive pulmonary disease (COPD) is the third leading cause of death in the United States, and is a large and growing burden on health care [4]. While smoking is the primary risk factor for rapid lung function decline and development of COPD, about 15% of individuals who have never smoked develop COPD and not all smokers succumb, implicating other factors, such as genetic, dietary, and lifestyle factors, in lifetime lung function patterns and disease risk [5].

Recent evidence indicates that vitamin D, as a steroid hormone capable of influencing gene expression, may be a determinant of lung function [6]. A cross-sectional study in the National Health and Nutrition Examination Survey (NHANES) III reported a strong positive association between serum 25(OH)D and lung function, with clinically relevant effect sizes for forced expiratory volume in the first second (FEV_1_) and forced vital capacity (FVC) [7]. However, a subsequent cross-sectional study in the U.K. reported no association between serum 25(OH)D and FEV_1_[8]. Causal inferences are limited in the cross-sectional design, effect estimates may be biased by uncontrolled confounders such as physical activity, and, furthermore, comparisons are limited by differences in the range in serum 25(OH)D between studies. Investigations of serum 25(OH)D or high-dose vitamin D supplementation in relation to the risk of exacerbations in COPD patients reported overall null findings [9,10]. However, vitamin D supplementation led to a statistically significant reduction in COPD exacerbations in the subgroup with severe vitamin D deficiency at the study baseline (serum 25(OH)D < 10 ng/mL) [9], underscoring the importance of considering the potential to benefit in studies of nutritional supplementation.

*In vitro* animal and cell culture studies demonstrate that vitamin D-responsive genes play a role in airway remodeling and inflammation, which are key processes in the pathogenesis of COPD [11,12]. However, few studies directly investigate mechanisms for vitamin D’s effect *in vivo,* which would strengthen the causal inference of population-level association studies. Furthermore, most experimental work to date has focused on effects of the active metabolite of vitamin D, 1,25-dihydroxyvitamin D. This metabolite is generated in the kidney for systemic circulation, and in many tissues, including lung [13]. It is not yet established whether the population-level range in serum 25-hydroxyvitamin D, the primary biomarker for vitamin D status in humans, is associated with effects similar to those seen *in vitro* for 1,25-hydroxyvitamin D.

We used an interdisciplinary approach to investigate the mechanisms through which vitamin D affects lung function. Genes with *in vitro* evidence of vitamin D regulation were studied to assess whether serum 25(OH)D concentration was associated with gene expression in lung epithelial tissues sampled from free-living humans. Identified genes were investigated in a study of expression quantitative trait loci (eQTL) in human lung epithelial cells to assess if genetic variation affects gene expression. Also, identified genes were investigated in an epidemiologic cohort study in relation to pulmonary function phenotypes. We hypothesized that serum 25(OH)D affects expression of vitamin D-responsive genes by modulating levels of active 1,25(OH)_2_D in lung tissue, and that variants in candidate genes directly regulated by 1,25(OH)_2_D in lung tissue are associated with FEV_1_ and FEV_1_/FVC, the key parameters used for COPD diagnosis and staging.

## Methods

### Gene expression study

Twenty-six healthy nonsmoker adult volunteers (Additional file 1) were recruited and evaluated at the Weill Cornell Medical College General Clinical Research Center under protocols approved by the Weill Cornell Medical College Institutional Review Board, as described elsewhere [14]. Frozen sera samples were assayed for 25(OH)D by liquid chromatography-tandem mass spectrometry at the Division of Laboratory Sciences, Centers for Disease Control and Prevention (Atlanta, GA). Airway epithelial cells were collected by brushing during bronchoscopy [14], and first and second strand cDNA were synthesized from 6 μg of RNA, *in vitro* transcribed, and fragmented according to Affymetrix protocols; samples were hybridized to the Affymetrix HG-U133 Plus 2.0 array [14]. (Additional file 2 for further details).

The microarray analysis considered 156 genes, which were identified *a priori* based on evidence of regulation by 1,25-dihydroxyvitamin D in squamous epithelial cells [1] and evidence for at least one predicted binding site for VDR (a DR3 or ER6 response element with up to 1 base mismatch from the consensus sequence) [1].

The statistical significance of fold-changes in expression between the first and third tertile of serum 25(OH)D was calculated using a t-test with Bayesian correction (Limma). Given that the purpose of the microarray study was to identify candidate genes to take forward to both the eQTL and the population-based cohort analysis, a statistical significance threshold of nominal P < 0.05 was used. Linear regression coefficients and the variance (R^2^) in gene expression explained by serum 25(OH)D were calculated, and included the full range of 25(OH)D concentrations.

### eQTL study: data collection and statistical approach

The Expression Quantitative Trait Loci (eQTL) study was conducted using lung small airway epithelium tissue samples from 116 individuals (see Additional file 2 for details). Tissue samples were collected under protocols approved by the Weill Cornell Medical College Institutional Review Board. Associations between SNPs and gene expression of 13 vitamin D-responsive genes in lung small airway epithelium tissue were analyzed. Tissue samples were taken from a diverse cohort of 116 smokers and non-smokers of different genders and ancestries (see Table 1, Gao *et al.*[15]). Details of the sample collection are published elsewhere [14] and details on normalization of gene expression values are available in Gao *et al.*[15] SNPs were assayed using Affymetrix 500 k arrays, which provided data on 191,959 genotypes; only SNPs with MAF of > 0.1 were analyzed for associations with gene expression. Thus, there were far fewer SNPs available in the eQTL study in comparison to the Health ABC GWAS study, and although very few of the exact SNPs studied in Health ABC were in the eQTL database, the eQTL SNPs tagged the sequence variation in each gene.

**Table 1:** Fold change in expression and P-value of 13 genes reaching nominal P-value Threshold (p < 0.05) in expression study

**Gene**	**Chromosome**	Fold change*	**P-value**	**R**^**2§**^
*KCNS3*	2	-1.62	0.00084	28%
*FSTL1*	3	-1.55	0.00163	40%
*DAPK1*	9	-2.06	0.00381	17%
*RSAD2*	2	1.41	0.01103	16%
*CST6*	11	1.79	0.01516	20%
*KAL1*	X	-1.38	0.01840	28%
*SLITRK6*	13	-1.52	0.02482	25%
*TMEM40*	3	1.55	0.02518	23%
*EMB*	5	1.52	0.03099	23%
*PTGER2*	14	1.36	0.03574	9%
*DTX4*	11	-1.34	0.03812	15%
*KLF4*	9	1.66	0.03901	9%
*SGPP2*	2	1.69	0.04491	24%

*Fold change in high versus low tertile serum 25-hydroxyvitamin D.

^§^R-squared calculated in linear regression, considering the full range of serum 25-hydroxyvitamin D, thus equals the proportion of variance in expression accounted for serum 25(OH)D.

SNPs within 100 kb of the 13 candidate genes (Additional file 3 for gene names) were tested for association with gene expression using PLINK v1.07. Quantile-quantile plots were generated in R and Locus Zoom [16] plots were generated to visually examine P-value distributions. The genome-wide Q-Q plot and Manhattan plot were also examined.

### Population-based cohort study

The Health, Aging and Body Composition (Health ABC) cohort study enrolled a random sample of European-Americans and all African-American Medicare-eligible residents, aged 70–79 at baseline (1997) and residing in the ZIP codes in and around Memphis, TN and Pittsburgh, PA (n = 3,075). The Institutional Review Boards at the University of Memphis, Tennessee, and the University of Pittsburgh granted approval to conduct the Health ABC Study. The Institutional Review Board at Cornell University and the Health ABC Publications Committee approved the use of Health ABC data for this study. The Framingham Heart Study (FHS) cohort (n = 7,694; includes individuals from the original, offspring, and third generation cohorts) [17] served as a replication cohort for cross-sectional SNP—lung function associations discovered in Health ABC European-Americans (Additional file 2 for further details on both cohort studies). The Institutional Review Board at the Boston University Medical Campus granted approval for the FHS.

Spirometry met American Thoracic Society criteria for acceptability [18,19]. Participants with missing covariate data were excluded from further consideration (~ 300 in each ancestry group). Participants with an FEV_1_ measurement and an FEV_1_/FVC ratio below the Lower Limit of Normal were considered to have prevalent airflow obstruction [19,20]. The Illumina Human 1 M-Duo custom chip was used for genotyping in Health ABC [21]. All assayed SNPs in the 13 candidate genes (identified by the expression study) with a minor allele frequency > 5% and in Hardy Weinberg equilibrium were analyzed, comprising 313 SNPs in European-Americans and 355 SNPs in African- Americans (Additional file 3).

Ordinary least squares linear regression models examined the relation between SNPs and FEV_1_ and FEV_1_/FVC in sequential regressions (using SAS 9.2). An additive genetic model was used to estimate the main effect of each SNP; SNPs with a nominal P ≤ 0.02 were further tested in dominant and recessive genetic models to refine effect estimates. In genetic studies, the risk of false positives must be minimized without ruling out true associations [22]. GWAS-scale multiple testing adjustments are not appropriate for the hypothesis-based investigation of the 13 genomic regions nominated by the gene expression study. Thus, SNPs with nominally significant p-values are presented, and False Discovery Rate (FDR) multiple testing correction was applied [23]. Models were adjusted for age, height, cigarette smoking (smoking status and pack-years), gender, study site, and ancestry principal components.

Sensitivity analyses were performed on the top findings for the FEV_1_ phenotype by repeating analyses after excluding individuals with prevalent airflow obstruction or individuals with lower quality spirometry (lower reproducibility scores). Exploratory SNP × serum 25(OH)D interaction analyses are presented in the additional file only (Additional files 4, 5).

## Results

### Gene expression by serum 25-hydroxyvitamin D

Healthy, non-smoking adults (n = 26) were divided into tertiles of serum 25(OH)D (range of serum 25(OH)D: 2.3-39.7 ng/mL); the lowest tertile boundary corresponded to the cutpoint for deficiency (< 12 ng/mL), and the upper tertile included only vitamin D sufficient individuals (all ≥ 20 ng/mL), thus further analysis compared these two groups. Expected associations were confirmed; serum vitamin D concentrations were lower in African American participants, and slightly higher in males (Additional file 1).

Among the 156 genes studied, thirteen genes (8.3%) had statistically significant (nominal p < 0.05) differences in expression between the first and third tertiles of serum 25-hydroxyvitamin D (Table 1). To further characterize the relation of serum 25-hydroxyvitamin D with the 13 nominally significant genes, the linear association of gene expression with continuous serum 25-hydroxyvitamin D was estimated (Table 1); the percent of variance (R^2^, from linear regression) explained by serum 25-hydroxyvitamin D ranged from 8 to 40%, and *FSTL1* had the highest R^2^.

### eQTL analysis

**Figure 1:**
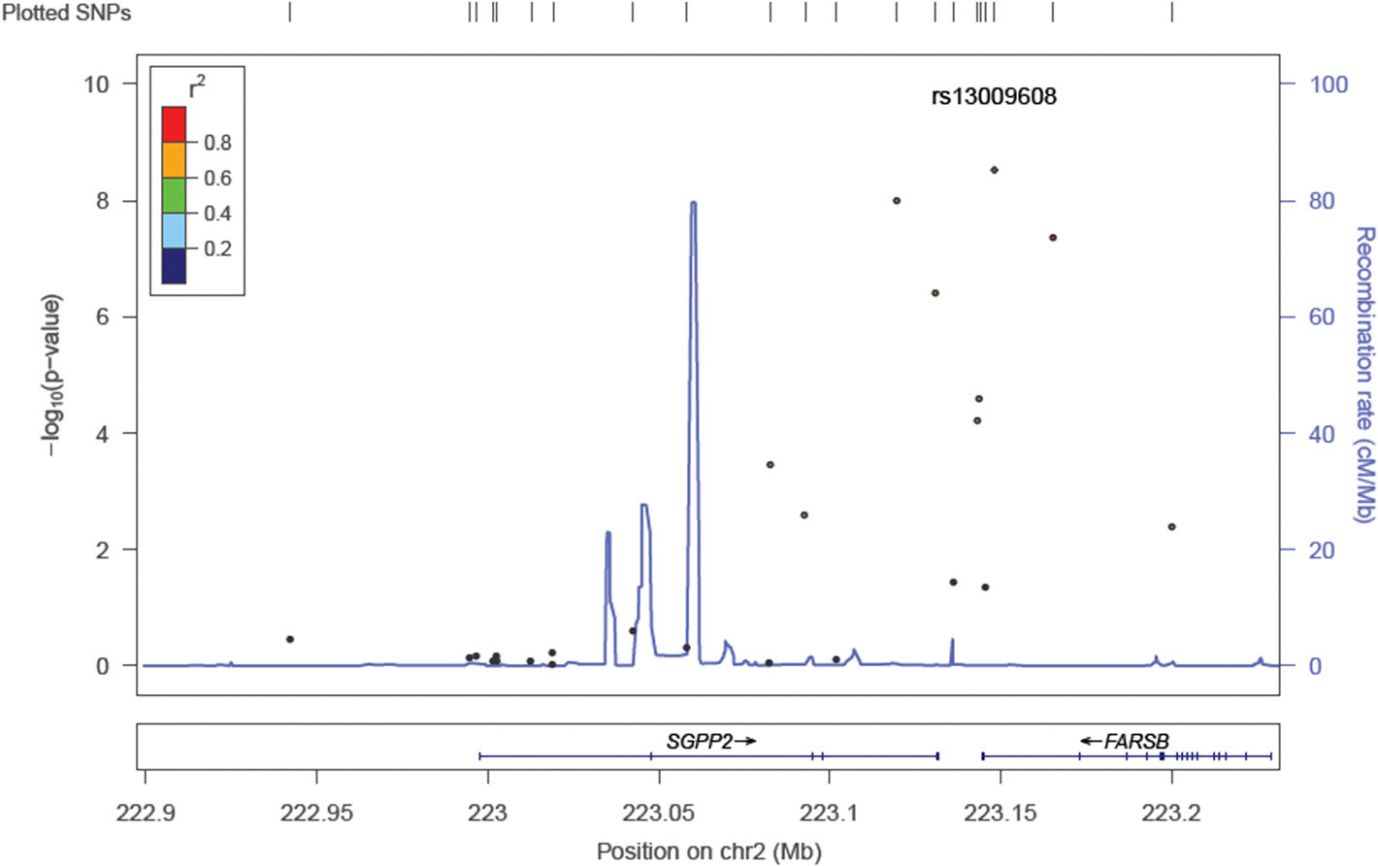
**Locus Zoom plot of *SGPP2 *** **eQTL associations.**

All 13 vitamin D-responsive genes were queried in the eQTL data, but only 12 genes had available data (no data for *RSAD2*). A highly statistically significant *cis* eQTL reaching genome-wide significance thresholds was identified for *SGPP2*; a cluster of SNPs in the 3’ region of *SGPP2* was associated with *SGPP2* gene expression in lung tissue (the lead SNP, rs13009608 had a nominal p-value of 2.99 × 10^-09^). Figure 1 shows gene-level results and Additional files 6 and 7 show genome-wide Q-Q and Manhattan plots, respectively. The association of rs13009608 with *SGPP2* gene expression was replicated (p-value: 7.0 × 10^-18^) in a publically available eQTL database of lymphoblastoid cell lines [24].

### Population-level SNP—lung function associations

All 13 vitamin D-responsive genes identified by the microarray screen were further studied in a population-based candidate gene association study. After excluding participants with missing covariate data, 1,502 European-Americans and 996 African-Americans (81% of full cohort) had an acceptable FEV_1_ and were included in the FEV_1_ analysis. 1,472 European-Americans and 943 African-Americans (79% of cohort) had an acceptable FEV_1_/FVC, and were included in the ratio analysis (Table 2).

**Table 2:** **Characteristics of Health, Aging and Body Composition study participants included in the FEV**_**1**_phenotype* analysis, stratified by race

**Covariate**	**African-Americans**	**European-Americans**
	**(N = 996)**	**(N = 1,502)**
Age, years**	73.4 (2.9)	73.7 (2.8)
Women (%)	553 (55.5)	708 (47.1)
Memphis, TN site (%)	464 (46.6)	759 (50.5)
Former Smokers (%)	398 (40)	746 (49.7)
Current Smokers (%)	167 (16.8)	99 (6.6)
Pack-years	29.5 (24.1)	36.5 (31.9)
FEV_1_, mL	1948.7 (569.4)	2305.4 (654.3)
FEV_1_/FVC	75.5 (9.3)	74.4 (7.9)
Height, cm	165.7 (9.4)	167 (9.3)
Mean 25(OH)D (ng/mL)***	20.9 (10.6)	29 (11)
COPD, defined by LLN (%)	66 (7.0)	110 (7.5)

*All participants in table have FEV_1_ data; approximately 50 fewer individuals have FEV_1_/FVC ratio data, but participant characteristics are the same for both phenotypes.

**Data shown are mean (SD) or number (%).

***Serum 25(OH)D measured for 1,412 (94%) European-Americans and 864 (87%) African-Americans with the FEV_1_ phenotype, and for 1,383 European-Americans and 864 African-Americans with the FEV_1_/FVC phenotype.

**Figure 2:**
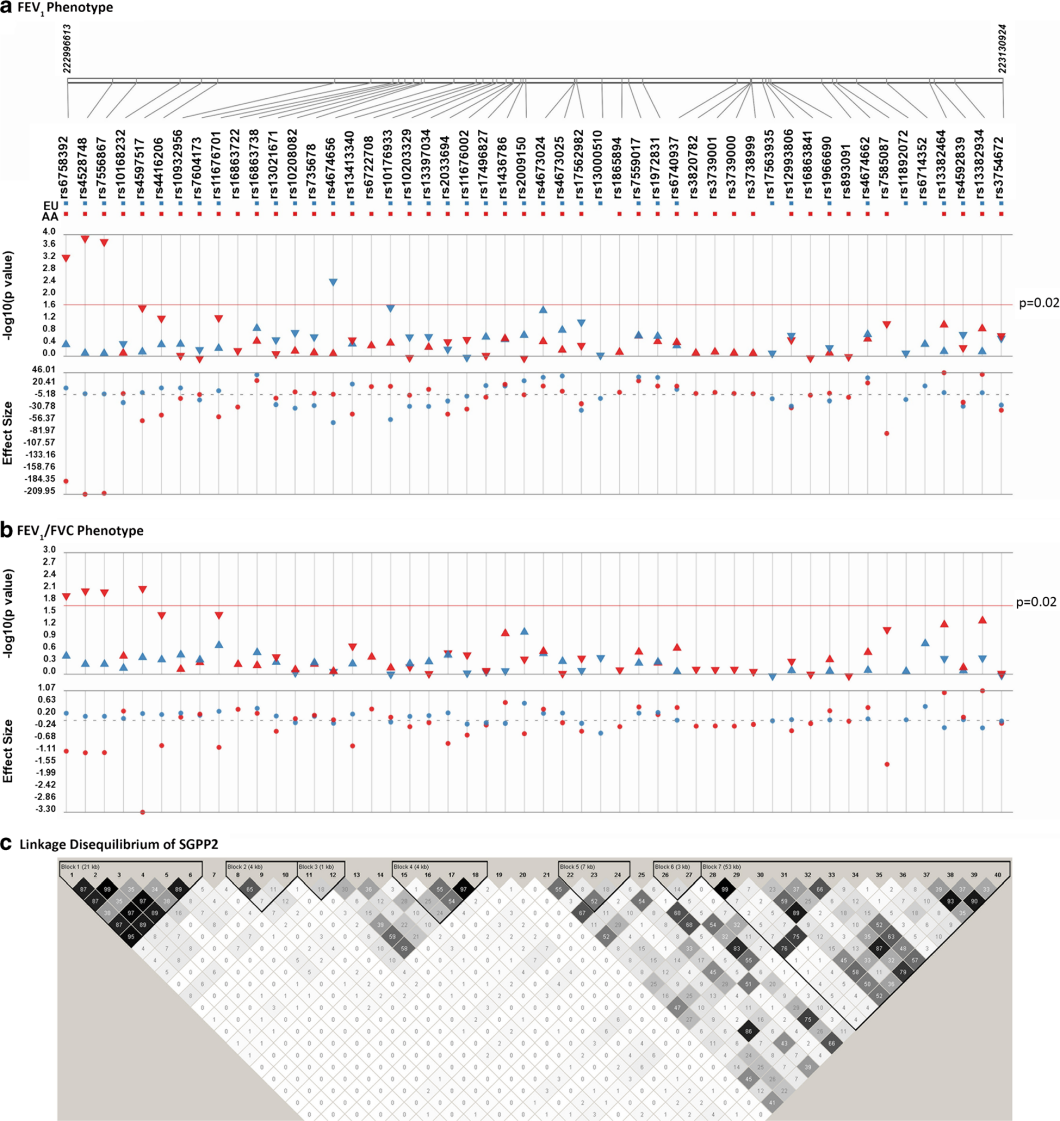
**Association between SNPs and FEV**_**1 **_**in *SGPP2*.** This figure shows all SNPs tested for association with FEV_1_ in African-Americans (red markers) and European-Americans (blue markers) in Health ABC. The top graph shows the p-values for each SNP on a negative log scale. The threshold for significance, nominal P = 2 × 10^-02^, is shown as a line in the figure. Effect estimates (β_SNP_) for FEV_1_ (in mL) for each ancestry group are shown underneath the P-values (dotted line shows null value of 0). Effect estimates and p-values are from recessive, dominant, or additive genetic models for SNPs with p < 0.02, and from an additive genetic model for all other SNPs. Finally, the linkage disequilibrium structure of *SGPP2* in the Health ABC European-American population is shown at the bottom, with darker shading representing higher R^2^.

Five SNPs in two genes (*DAPK1* and *SGPP2)* were associated with FEV_1_ at a nominal P < 0.02 in European-American participants (P-value range: 2.88 × 10^-03^ to 1.92 × 10^-02^; Table 3). A SNP in *DAPK1* (rs11141878) had the largest effect; participants with two copies of the minor allele (recessive genotype) were 104 mL lower on FEV_1_*.* In African-Americans, 18 SNPs in 6 genes (*DAPK1, FSTL1, KAL1, KCNS3, RSAD2,* and *SGPP2*) were associated with FEV_1_ at nominal P < 0.02 (range: 1.11 × 10^-04^ to 1.65 × 10^-02^; Table 4). A group of 3 linked SNPs in a linked 5’ block of *SGPP2* were associated with a decreased FEV_1_ and a reduced FEV_1_/FVC ratio in African-Americans with nominal P-values <0.02 and FDR q-values <0.05 (Figure 2). A fourth SNP in *SGPP2*, rs4597517, was borderline significantly associated with FEV_1_ in African-Americans in the additive model (p = 2.16 × 10^-2^), and statistically significantly associated with FEV_1_ (p = 4.28 × 10^-4^) in the recessive genetic model. A SNP in *KCNS3* (rs3747515) had the largest effect on FEV_1_ in African-Americans; participants with the recessive genotype were 244 mL higher on FEV_1_*.* Due to linkage, some SNP associations were redundant; thus, SNPs in the same gene with an R^2^ ≥ 0.9 (indicating strong linkage) are assumed to represent the same effect and redundant SNPs are presented in the online additional materials only (Additional files 8, 9).

**Table 3:** **The association of SNPs in vitamin D-responsive genes (nominal P < 2.0 × 10**^**-02**^**) with FEV**_**1**_(mL) for European-Americans in the Health, Aging and Body Composition study (sorted by gene)*

**Gene**	**RS#**	**Chr**	**Coded allele**	**MAF (%)**	β**	**SE**	**Nominal P**	**Model**
***DAPK1***	rs11141878	9	A	36	-103.98	36.3	4.26 × 10^-03^	R
	rs4877361†	9	G	14	72.47	27.4	8.17 × 10^-03^	D
	rs4878089	9	A	46	39.68	16.9	1.92 × 10^-02^	A
***SGPP2***	rs4674656	2	A	25	-58.70	19.7	2.88 × 10^-03^	A

†one redundant SNP not shown.

*Abbreviations: Chr, chromosome; MAF, minor allele frequency; β, regression coefficient; SE, standard error; A = additive genetic model, D = dominant model, R = recessive model.

**Beta-coefficient estimates the association of allele with FEV_1_, based on genetic model shown and adjusted for age, height, smoking, gender, study site, and ancestry principal components.

**Table 4:** **The association of SNPs in vitamin D-responsive genes (nominal P < 2.0 × 10**^**-02**^**) with FEV**_**1**_(mL) for African-Americans in the Health, Aging and Body Composition study (sorted by gene)*

**Gene**	**RS#**	**Chr**	**Coded allele**	**MAF (%)**	β**	**SE**	**Nominal P**	**Model**
***DAPK1***	rs3128491	9	G	33	51.48	21.4	1.65 × 10^-02^	A
***FSTL1***	rs4676781	3	T	8	-110.13	35.3	1.88 × 10^-03^	A
	rs13100865	3	G	9	-105.96	35.0	2.54 × 10^-03^	A
	rs13097755†	3	T	28	-60.46	21.6	5.20 × 10^-03^	A
***KAL1***	rs6530200	23	T	47	-45.28	16.8	7.20 × 10^-03^	A
	rs974655	23	A	49	79.23	30.3	9.14 × 10^-03^	D
***KCNS3***	rs1031771†	2	A	16	243.76	83.5	3.60 × 10^-03^	R
***RSAD2***	rs4669114††	2	G	10	-119.55	36.2	9.93 × 10^-04^	D
	rs6431837	2	C	47	-101.06	33.6	2.66 × 10^-03^	R
	rs7570384	2	C	38	-55.35	20.1	5.88 × 10^-03^	A
	rs4669111	2	A	41	-49.75	20.1	1.34 × 10^-02^	A
***SGPP2***	rs4528748††	2	C	27	-209.95	54.1	1.11 × 10^-04^***	R

*Abbreviations: Chr, chromosome; MAF, minor allele frequency; β, regression coefficient; SE, standard error; A = additive genetic model, D = dominant model, R = recessive model.

**Beta-coefficient estimates the association of allele with FEV_1_, based on genetic model shown, adjusted for age, height, smoking, gender, study site, and ancestry principal components.

***FDR q-value <0.05.

†one redundant SNP not shown.

††two redundant SNPs not shown.

In European-Americans, 1 SNP in *KLF4* was associated with the FEV_1_/FVC ratio (P-value 1.15 × 10^-2^; Additional file 9). In African-Americans, 14 SNPs in 3 genes (*FSTL1, KAL1,* and *SGPP2*) were associated with the ratio at a nominal P < 0.02 (range: 1.32 × 10^-03^ to 1.27 × 10^-02^; Additional file 9).

A sensitivity analysis explored whether the SNP—FEV_1_ associations primarily reflected effects of genetic variation on risk of COPD; analyses were repeated after excluding 110 European Americans and 64 African-Americans with prevalent airflow obstruction (as an indicator of COPD). For European-Americans there was little or no difference in analyses with and without prevalent cases. A Bland-Altman analysis showed that for SNPs in *SGPP2,* the effect estimates for African-Americans were attenuated after excluding cases of prevalent airflow obstruction (data not shown). Thus, the *SGPP2* SNPs that had statistically significant associations with FEV_1_ were further tested in logistic regression models to assess the *SGPP2—*outcome association in African-Americans. Individuals with two copies of the SNP most statistically significantly associated with FEV_1_, rs4528748, had a 2.6-fold increased risk of airflow obstruction. All 3 *SGPP2* SNPs had odds ratios above 2 for the SNP—COPD association, and all confidence intervals excluded 1 (Table 5), supporting a role for *SGPP2* in mediating COPD risk.

**Table 5:** **Associations of SNPs in *SGPP2 *** with risk of prevalent COPD* in African-Americans in the Health, Aging and Body Composition Study

		**95% Confidence interval**		
SNP**	**Odds ratio**	**Lower**	**Upper**	**Nominal P-value**
rs4528748	2.63	1.19	5.80	1.64 × 10^-02^
rs7556867	2.71	1.23	5.99	1.35 × 10^-02^
rs6758392	2.34	1.07	5.11	3.33 × 10^-02^

*COPD defined as FEV_1_ and FEV_1_/FVC ratio below the Lower Limit of Normal.

*All SNPs modeled as recessive (two copies of the minor allele) to reflect the most significant coding from Table 3, and models adjusted for age, height, smoking, gender, study site, and ancestry principal components.

There was consistency of findings across both phenotypes and both ancestry groups for 2 genes, namely *SGPP2* and *DAPK1*. SNPs in *SGPP2* and *DAPK1* were associated with FEV_1_ in both European-Americans and African-Americans, and SNPs in *SGPP2* were also associated with FEV_1_/FVC and with risk of prevalent airflow obstruction in African-Americans.

Genes containing SNPs significantly associated with FEV_1_ or FEV_1_/FVC in Health ABC European-Americans, namely *DAPK1, KLF4,* and *SGPP2*, were further evaluated in the FHS cohort. Gene-level replication was observed for *DAPK1* and *SGPP2;* 23 out of 340 SNPs in *DAPK1* (6.8%) and 23 out of 145 SNPs (15.8%) in *SGPP2* were associated with cross-sectional FEV_1_ at a nominal P-value <0.05 in the FHS cohort, although these comprised different SNPs than the ones associated with lung function in Health ABC (Additional file 10).

## Discussion

Using an interdisciplinary genomics approach we investigated vitamin D and lung outcomes. *SGPP2,* a phosphatase involved in the sphingosine-1-phosphate signaling pathway, was identified in all stages of the study as a promising candidate gene contributing to vitamin D-mediated associations with lung function. *SGPP2* is differentially expressed *in vivo* in lung epithelial cells by serum 25(OH)D. eQTL analysis demonstrates that sequence variants in *SGPP2* are associated with lung cell gene expression. Although the eQTL finding does not prove that vitamin D regulation affects gene expression, the location of associated variants in regulatory regions supports the hypothesis of vitamin D regulation. Furthermore, a group of 3 linked SNPs in the *SGPP2* promoter region are associated with lower FEV_1_, a reduced FEV_1_/FVC ratio, and a 2–3 fold increased risk of airflow obstruction in African-Americans, suggesting that a causal variant in this region may affect *SGPP2* function and/or vitamin D binding, and, consequently, lung outcomes. Additionally, a SNP in *SGPP2* is associated with FEV_1_ in Health ABC European-Americans and *SGPP2* variants were also associated with FEV_1_ in the Framingham Heart Study, confirming effects across racial groups and in two cohort studies. This multi-faceted approach identifies putative mechanistic pathways for observed vitamin D—lung function associations while reducing the chance of false positive results.

*SGPP2* plays a key role in the sphingolipid signaling pathway through dephosphorylation of sphingosine-1-phosphate (S1P) to sphingosine, which is then converted to ceramide or back to sphingosine-1-phosphate by other enzymes [25]. Sphingosine-1-phosphate acts as both an intracellular and extracellular signaling molecule, and regulates critical cell processes including apoptosis, cell growth, and immune function [25,26]. Altered sphingolipid concentrations have important ramifications for lung function; ceramide concentrations are elevated in COPD, contributing to lung alveolar destruction [25]. Little research exists on *SGPP2,* although a 2006 paper showed that *SGPP2* is up-regulated in response to inflammatory stimuli in endothelial cells, suggesting a possible role in mediating inflammation in lung tissue [27]. However, *SGPP2*’s biological function to alter sphingosine-1-phosphate concentrations suggests that this gene contributes to the regulation of sphingolipid signaling pathways in lung tissue.

We identified several additional genes, namely *DAPK1, KCNS3,* and *FSTL1,* and all three had mechanistic links to lung function identified through gene ontology analysis and literature reviews (Additional files 11 and 12). Expression of all three genes was strongly associated with serum 25(OH)D, and variants in these genes were associated with pulmonary function in the Health ABC cohort study. However, variants were not replicated in the Framingham Heart Study, nor were there observed eQTL associations. *DAPK1,* which is down-regulated by 1,25(OH)_2_D both *in vivo* and *in vitro,* is a pro-apoptotic kinase linked to cytoskeletal remodeling and regulation of inflammatory gene expression in macrophages [28,29]. SNPs in *KCNS3,* which encodes a voltage-gated potassium channel protein, were associated with airway hyperresponsiveness in past studies [30], which is of interest given postulated associations of airways hyperresponsiveness with an accelerated rate of FEV_1_ decline and risk of COPD [31]. *FSTL1* up-regulates pro-inflammatory cytokines; in mice, the highest expression level is in lung [32]. Dexamethasone, which is a glucocorticoid used to treat both asthma and COPD, is associated with expression of both *KCNS3* and *FSTL1;* interestingly, there are striking similarities in the effects of dexamethasone and 1,25-dihydroxyvitamin on the expression of these genes. The combination of 1,25-dihydroxyvitamin D with dexamethasone was investigated *in vitro* as an anti-inflammatory treatment; our results suggest the strong possibility of synergistic effects for this treatment combination (Additional file 12 for references).

A major strength of this study is that it translates *in vitro* animal and cell culture studies to an *in vivo* study, and then extends to study population-level SNP associations with lung phenotypes, which are partially replicated in an independent cohort. The multi-stage approach identified *SGPP2* as a promising vitamin D-responsive gene for further study. The demonstration of differential gene expression in lung tissue associated with the physiologic range of 25-hydroxyvitamin D in a diverse sample of free-living humans confirms *in vitro* studies, and, while our study does not manipulate vitamin D, the *in vivo* evidence of association is novel. The Health ABC population-based cohort study included high-quality spirometry, detailed information on confounding factors such as smoking and population stratification, and comprised 40% African-American participants, thus allowing consideration of this understudied population in genomic research. FEV_1_ is a predictor of all-cause mortality [33], and thus SNP—FEV_1_ associations are clinically relevant. Although associations between SNPs and the FEV_1_/FVC ratio were also investigated, the associations were not as strong as for FEV_1_. Thus, vitamin D may have a stronger association with overall lung health versus the risk of COPD. This study identifies plausible biological mechanisms that support a true effect of vitamin D on lung function, and will help to guide the design and analysis of randomized controlled intervention trials of the role of vitamin D in lung disease.

Given that the microarray analysis was used exclusively as a candidate screen, limitations including the lack of qPCR confirmation (not possible due to sample volume limitations), use of nominal P values, and the lack of race-stratified analysis (not possible due to sample size limitations) are less of a concern. As expected, the proportion of participants in the race/ethnicity groups varied by tertile of serum 25(OH)D given the role of skin pigmentation in vitamin D synthesis in response to sunlight [2]. Race may either confound the serum 25(OH)D—gene expression association, or, race may be a causal antecedent variable that, in part, causes serum 25(OH)D concentration and, in turn, differences in gene expression; adjusting for race may be an over-adjustment. Of note, in regressions adjusted for race the regression coefficients for the serum 25(OH)D—gene expression association were similar to unadjusted analyses.

While the studies were all cross-sectional, which limits causal inference, the harmony of findings across different designs partly mitigates this concern. Although it would have been ideal to use the same samples in all studies (that is, expression, eQTL and SNP—lung function studies), practical limitations led to the use of different samples in each phase. Finally, although gene-level replication was observed for *SGPP2* and *DAPK1*, the specific SNPs associated with FEV_1_ in Health ABC did not reach statistical significance in FHS. We hypothesize that the *SGPP2* SNPs identified in the two cohort studies may be tagging the same unknown causal variant(s) or there may be multiple *SGPP2* regulatory regions associated with lung function. Additionally, the strongest SNP—lung function associations in Health ABC were in African-Americans, and, because FHS includes only European Americans, the replication was partial. In summary, SNPs in *SGPP2* were statistically significantly associated with lung outcomes after FDR multiple testing adjustment and a highly statistically significant lung eQTL was identified for *SGPP2*; *SGPP2* emerged as a clear candidate in all stages of this work.

## Conclusions

This study establishes for the first time that physiological concentrations of serum 25(OH)D are associated with differences in gene expression in human lung tissue, and that candidate vitamin D responsive genes are associated with pulmonary function outcomes. We hypothesize that genetic variants associated with pulmonary function in our study affect binding of the VDR/RXR heterodimer to the genome; however, further studies are needed to map lung tissue-specific regulatory regions. Recent evidence shows that vitamin D regulatory elements (VDREs) are located both proximal and distal to vitamin D-responsive genes at promoter regions and enhancer regions, respectively, and that VDR/RXR binding is cell-type specific [34]. This emphasizes the importance of genome-wide VDR/RXR mapping in lung cells to identify regulatory regions [34]. Additionally, *in vitro* studies of bronchial epithelial cells to directly assess gene expression changes due to vitamin D would contribute to the current understanding. Overall, the results of our study identify putative mechanisms through which vitamin D may affect lung function and, suggest a physiological range for 25-hydroxyvitamin D at which differential responses occur at the molecular level. Demonstrated associations strengthen the evidence for monitoring serum 25(OH)D concentrations in individuals at risk of rapid decline in lung function.

## Abbreviations

COPD: Chronic obstructive pulmonary disease; FEV1: Forced expiratory volume in the first second; FDR: False discovery rate; FVC: Forced vital capacity; 25(OH)D: 25-hydroxyvitamin D; 1,25(OH)2D: 1,25-dihydroxyvitamin D; NHANES: National Health and Nutrition Examination Survey; CST6: cystatin E/M; DAPK1: Death associated protein kinase 1; DTX4: Deltex homolog 4; EMB: Embigin; FSTL1: Follistatin-like 1; KAL1: Kallmann syndrome 1 sequence; KCNS3: Potassium voltage-gated channel, delayed-rectifier, subfamily S, member 3; KLF4: Kruppel-like factor 4; PTGER2: Prostaglandin E receptor 2(subtype EP2); RSAD2: Radical S-adenosyl methionine domain containing 2; SLITRK6: SLIT and NTRK-like family, member 6; SGPP2: Sphingosine-1-phosphate phosphatase 2; TMEM40: Transmembrane protein 40.

## Competing interests

The authors declare that they have no competing interests.

## Authors’ contributions

All authors satisfy the requirements for authorship and contributorship. BR, RC and PAC designed and conducted the expression study; YL, KL, SK and TH conducted the Health ABC GWAS study, which provided data for this paper; JGH, BR and PAC designed the Health ABC SNP study and JGH and PAC conducted the SNP study; JGH, PAC, JW and GO'C conducted the replication analysis in FHS, and JGH, PAC, JM and CG conducted the eQTL analysis and interpretation. All coauthors read and edited the final manuscript.

## Pre-publication history

The pre-publication history for this paper can be accessed here:

http://www.biomedcentral.com/1471-2350/14/122/prepub

## Supplementary Material

Additional file 1: Table S1 Characteristics of 26 Non-smoking Human Volunteers in the Gene Expression Study, by Tertile of Serum 25-Hydroxyvitamin D Concentration. Click here for file

Additional file 2 Methods and Results. Click here for file

Additional file 3: Table S2 The distribution of studied SNPs in Thirteen Vitamin D-responsive Genes for European- and African-American Ancestry Groups in the Health ABC Cohort Study. Click here for file

Additional file 4: Table S3 SNP by 25(OH)D interactions associated with the FEV_1_ phenotype in a) European-Americans, and b) African-Americans. Click here for file

Additional file 5: Table S4 SNP by serum 25(OH)D interactions in association with the FEV_1_/FVC phenotype in a) European-Americans, and b) African-Americans. Click here for file

Additional file 6: Figure S1 Genome-wide Quantile-Quantile Plot for *SGPP2* eQTL findings. Click here for file

Additional file 7: Figure S2 Genome-wide Manhattan Plot for *SGPP2* eQTL findings. Click here for file

Additional file 8: Table S5 The most statistically significant associations (nominal P < 2.0 × 10^-02^) between single nucleotide polymorphisms in vitamin D-responsive genes and FEV_1_ for a) European-Americans and b) African-Americans (all SNPs, including redundant SNPs are shown). Click here for file

Additional file 9: Table S6 The most statistically significant associations (nominal P < 2.0 × 10^-02^) between single nucleotide polymorphisms in vitamin D-responsive genes and the FEV_1_/FVC ratio for a) European-Americans and b) African-Americans in the Health ABC cohort. Click here for file

Additional file 10: Table S7 Gene-level replication of Health ABC European-American SNP associations with FEV_1_ using the Framingham Heart Study cohort. Click here for file

Additional file 11: Table S8 Gene Ontology of Thirteen Nominally Significant Candidate Genes from the UniProtKb-GOA Database (http://www.ebi.ac.uk/QuickGO/). Click here for file

Additional file 12: Table S9 Evidence Supporting the Role in Lung Health and/or Regulation by Glucocorticoids For Genes Differentially Expressed by Serum Vitamin D. Click here for file
