# Supplementary material for: Vitamin D-responsive SGPP2 variants associated with lung cell expression and lung function
Source: BMC Med Genet. 2013 Nov 25;14:122. doi: 10.1186/1471-2350-14-122 (PMC3907038; doi:10.1186/1471-2350-14-122)
Supplement: Additional file 1: Table S1 — Characteristics of 26 Non-smoking Human Volunteers in the Gene Expression Study, by Tertile of Serum 25-Hydroxyvitamin D Concentration. [file 1471-2350-14-122-S1.docx]

**Additional file 1: Table S1.** Characteristics of 26 Non-smoking Human Volunteers in the Gene Expression Study, by Tertile of Serum 25-Hydroxyvitamin D Concentration

|  | **Serum 25-Hydroxyvitamin D** | | |
| --- | --- | --- | --- |
| **Variable** | **Tertile I (n=9)** | **Tertile II (n=9)** | **Tertile III (n=8)** |
| Serum 25-OH-D,  ng/mL (range) | 8.99 (2.3 - 11.8) | 20.9 (12.7 - 26.7) | 33.3 (27.9 - 39.7) |
| Age, years (median) | 36.9 (38) | 44.1 (45) | 50.6 (46.5) |
| Males (%) | 6 (67%) | 6 (67%) | 7 (87%) |
| Race/Ethnicity (%)  African American | 5 (56%) | 6 (67%) | 1 (13%) |
| European | 1 (11%) | 3 (33%) | 7 (87%) |
| Hispanic | 2 (22%) | 0 (0%) | 0 (0%) |
| Asian | 1 (11%) | 0 (0%) | 0 (0%) |

*mean (standard deviation), unless noted
