# Supplementary material for: Vitamin D-responsive SGPP2 variants associated with lung cell expression and lung function
Source: BMC Med Genet. 2013 Nov 25;14:122. doi: 10.1186/1471-2350-14-122 (PMC3907038; doi:10.1186/1471-2350-14-122)
Supplement: Additional file 2 — Methods and Results. [file 1471-2350-14-122-S2.docx]

**Additional file 2**

**METHODS**

**Gene Expression Study: Serum 25(OH)D Assays**

Frozen serum samples from the 26 individuals recruited for the gene expression study were sent to the Division of Laboratory Sciences at the Centers for Disease Control and Prevention to be assayed for 25(OH)D by liquid chromatography-tandem mass spectrometry. Samples were evaluated in parallel with NIST standard SRM 972, and results were within 2 standard deviations of NIST target values. External quality assurance was provided through participation in the Vitamin D External Quality Assessment Scheme.

Two individuals were excluded because serum and cell collections occurred in different seasons and more than 120 days apart (their serum measurements were in the second tertile of the distribution). The average time between serum and epithelial cell collection was 42 ± 40 days, within the 10-week half-life of serum 25-hydroxyvitamin D.

**Gene Expression Study: Microarrays**

Image files for the arrays were assessed for quality of hybridization by comparing 3’ to 5’ intensity of transcripts for actin and GAPDH (ratio < 3). Normalization was carried out by GeneChip Robust Multi-Array Average using MADMAX software (https://madmax.bioinformatics.nl). Quality control of normalized data was evaluated using plots of relative log expression and normalized unscaled standard errors to identify array artifacts. Only probe sets with an interquartile range (IQR) of log_2_ normalized values < 0.5 were included in analysis.

**Gene Expression Study: Gene Ontology Analysis**

Gene ontology annotations were obtained from the UniProtKb-GOA database (http://www.ebi.ac.uk/QuickGO/), with preference given to IDA annotations (inferred from direct assay) or TAS (traceable author statement) evidence codes. IEA (inferred from electronic annotation) evidence codes were used if no other information was available.

**Population-based Cohort Study: Participants, Data Collection and Statistical Approach**

Participants: To be eligible for the Health ABC cohort study, participants were required to be ambulatory, that is, to have no difficulty walking ¼ mile or climbing 10 stairs without resting, and to be able to independently perform basic activities. Additionally, participants were required to have no history of active cancer treatment at baseline, and no plans to leave the area within 3 years after study baseline. A total of 3,075 individuals were enrolled.

Data Collection: Spirometry was conducted by trained personnel using a dry rolling seal spirometer (SensorMedics Corporation, Yorba Linda, CA) connected to a personal computer. Pulmonary function tests from the baseline clinic visit meeting American Thoracic Society (ATS) criteria for acceptability were included in this study.[[1](#_ENREF_1)]

Statistical Approach: In all statistical models, SNPs were coded as the number of minor alleles an individual had at a specific genetic locus, based on Health ABC-specific allele frequency data. All models adjusted for population substructure using principle components, which were computed separately by race across all markers. Redundancy between SNP associations was assessed using SNAP[[2](#_ENREF_2)] in CEU and YRI HapMap populations. SNP-pulmonary function associations were visualized using SynthesisView[[3](#_ENREF_3)], and linkage disequilibrium in the Health ABC cohort was evaluated in Haploview 4.2.

In a sensitivity analysis to explore the effect of spirometry quality on the findings, individuals with acceptable tests that were lower quality by reproducibility criteria were excluded and the SNP—FEV_1_ association was assessed in the subset. The direction and magnitude of all effect estimates were similar to the full sample results (data not shown).

**Population-based Cohort Study: Gene-Environment Interaction Analyses**

Serum 25(OH)D measurements were completed on a majority of Health ABC participants, which allowed the consideration of gene by nutrient interaction. Serum 25(OH)D was measured in stored serum samples from the 12-month follow-up visit using a 2-step radioimmunoassay (25-Hydroxyvitamin D 125I RIA Kit, DiaSorin, Stillwater, Minn., USA); the interassay coefficient of variation was 6.8% for log transformed 25(OH)D values.[[4](#_ENREF_4)] Although the serum 25(OH)D measurements are from the 12-month follow-up visit, a recent study reported a correlation of 0.8 between vitamin D measurements taken a year apart[[5](#_ENREF_5)], supporting the assumption that measured 25(OH)D is an excellent representation of vitamin D serum status at study baseline.

Genotype by serum 25(OH)D interactions were assessed in an additive model by including a product term between each SNP and serum 25(OH)D, adjusting for season of vitamin D measurement; a less stringent nominal P-value threshold (P<0.05) was used for interaction analyses because of lower power to detect effects. In gene-nutrient interaction analyses, participants with missing serum 25(OH)D data were excluded.

**Population-based Cohort Study: Replication in Framingham Heart Study**

The Framingham Heart Study (FHS) cohort (n=7,694; includes individuals from the original, offspring, and third generation cohorts) was used as a replication cohort to examine the genes with cross-sectional SNP associations (nominal p<0.02) in Health ABC European-Americans. FHS genotyping used the Affymetrix GeneChip Human Mapping 500K Array and 50K Human Gene Focused Panel[[6](#_ENREF_6)].

**eQTL Analysis: Data Collection and Statistical Approach**

Associations between SNPs and gene expression of 13 vitamin D-responsive genes in lung small airway epithelium tissue were analyzed. Tissue samples were taken from a diverse cohort of smokers and non-smokers of different genders and ancestries (see Table 1, Gao *et al*[[7](#_ENREF_7)]). Details of the sample collection are published elsewhere,[[8](#_ENREF_8)] and details on normalization of gene expression values are available in Gao *et al.*[[7](#_ENREF_7)] SNPs were assayed using Affymetrix 500k arrays, which provided data on 191,959 genotypes; only SNPs with MAF of > 0.1 were analyzed for associations with gene expression. Thus, there were far fewer SNPs available in the eQTL study in comparison to the Health ABC GWAS study, and although very few of the exact SNPs studied in Health ABC were in the eQTL database, the eQTL SNPs tagged the sequence variation in each gene.

SNPs within 100kb of the 13 candidate genes (Supplemental Table 3 for gene names) were tested for association with gene expression using PLINK v1.07. Quantile-quantile plots were generated in R and Locus Zoom[[9](#_ENREF_9)] plots were generated to visually examine P-value distributions. Genome-wide Q-Q plot and Manhattan plot were also examined.

**RESULTS**

**Population-based Cohort Study: Gene-Environment Interaction Analyses**

Genotype by serum 25(OH)D interactions were investigated for the FEV_1_ and FEV_1_/FVC phenotypes in both groups to assess the evidence that genotype—phenotype associations varied by vitamin D. In European-Americans, the genotype—FEV_1_ association was modified by serum 25(OH)D for 10 SNPs in 4 genes (*DAPK1, KAL1, SGPP2,* and *SLITRK6)* at nominal P<5.0x10^-02^. In African-Americans, the genotype—FEV_1_ association was modified by serum 25(OH)D for 43 SNPs in 9 genes (*DAPK1, DTX4, EMB, FSTL1, KAL1, KCNS3, PTGER2, SGPP2, SLITRK6)* at nominal P<5.0x10^-02^.

In European-Americans, the genotype—FEV_1_/FVC association was modified by serum 25(OH)D for 11 SNPs in 4 genes (*KAL1, PTGER2, SGPP2,* and *TMEM40*) at nominal P<5.0x10^-02^. In African-Americans, the genotype—FEV_1_/FVC association was modified by serum 25(OH)D for 26 SNPs in 8 genes (*DAPK1, DTX4, EMB, FSTL1, KAL1, KCNS3, PTGER,* and *SGPP2*) at nominal P<5.0x10^-02^ (Additional File 10).

The *SGPP2*—lung function association was consistently modified by serum 25(OH)D. Thus, the SNP—phenotype association for SNPs in the *SGPP2* gene was modified by serum 25(OH)D in European- and African-Americans for both the FEV_1_ and the FEV_1_/FVC phenotypes, with consistent direction of effect for the interaction effect (higher serum vitamin D concentrations attenuated genotype—phenotype associations). Similarly, SNPs in *DAPK1, DTX4, EMB, FSTL1, KAL1,* and *PTGER2* showed consistent evidence of genotype—serum 25(OH)D interactions for both FEV_1_ and FEV_1_/FVC in African-Americans.

For statistically significant genotype x serum 25(OH)D interactions (nominal P<5.0x10^-02^) the mean FEV_1_ was estimated (from model coefficients) for serum 25(OH)D concentrations of 12 ng/mL, 20 ng/mL, and 30 ng/mL, corresponding to typical definitions of deficient, sufficient, and optimal levels of vitamin D nutriture, respectively. At each level of serum 25(OH)D, individuals with 0 copies of the variant allele (wild-type homozygotes) were compared to individuals with 1 or 2 copies of the variant allele. In European-American participants, gene x nutrient interactions were consistent such that the allele—FEV_1_ association was stronger at the “deficient” level of serum 25(OH)D (12 ng/ml). In African-Americans, findings were mixed; interaction results went in both directions (Additional File 10). The pattern of findings for the ratio phenotype is similar (Additional File 11).

REFERENCES

1. Waterer GW, Wan JY, Kritchevsky SB, Wunderink RG, Satterfield S, Bauer DC, Newman AB, Taaffe DR, Jensen RL, Crapo RO: Airflow limitation is underrecognized in well-functioning older people. *J Am Geriatr Soc* 2001, 49(8):1032-1038.

2. Johnson AD, Handsaker RE, Pulit SL, Nizzari MM, O'Donnell CJ, de Bakker PI: SNAP: a web-based tool for identification and annotation of proxy SNPs using HapMap. *Bioinformatics* 2008, 24(24):2938-2939.

3. Barrett JC: Haploview: Visualization and analysis of SNP genotype data. *Cold Spring Harb Protoc* 2009, 2009(10):pdb ip71.

4. Shea MK, Houston DK, Tooze JA, Davis CC, Johnson MA, Hausman DB, Cauley JA, Bauer DC, Tylavsky F, Harris TB *et al*: Correlates and prevalence of insufficient 25-hydroxyvitamin D status in black and white older adults: the health, aging and body composition study. *J Am Geriatr Soc* 2011, 59(7):1165-1174.

5. Jorde R, Sneve M, Emaus N, Figenschau Y, Grimnes G: Cross-sectional and longitudinal relation between serum 25-hydroxyvitamin D and body mass index: the Tromso study. *European journal of nutrition* 2010, 49(7):401-407.

6. Hancock DB, Eijgelsheim M, Wilk JB, Gharib SA, Loehr LR, Marciante KD, Franceschini N, van Durme YM, Chen TH, Barr RG *et al*: Meta-analyses of genome-wide association studies identify multiple loci associated with pulmonary function. *Nat Genet* 2010, 42(1):45-52.

7. Gao C, Tignor N, Salit J, Strulovici-Barel Y, Hackett N, Crystal RG, Mezey JG: HEFT: eQTL analysis of many thousands of expressed genes while simultaneously controlling for hidden factors. *Manuscript submitted for publication*.

8. Harvey BG, Heguy A, Leopold PL, Carolan BJ, Ferris B, Crystal RG: Modification of gene expression of the small airway epithelium in response to cigarette smoking. *J Mol Med (Berl)* 2007, 85(1):39-53.

9. Pruim RJ, Welch RP, Sanna S, Teslovich TM, Chines PS, Gliedt TP, Boehnke M, Abecasis GR, Willer CJ: LocusZoom: regional visualization of genome-wide association scan results. *Bioinformatics* 2010, 26(18):2336-2337.

10. Cheng BH, Liu Y, Xuei X, Liao CP, Lu D, Lasbury ME, Durant PJ, Lee CH: Microarray studies on effects of Pneumocystis carinii infection on global gene expression in alveolar macrophages. *BMC Microbiol* 2010, 10:103.

11. Miyamae T, Marinov AD, Sowders D, Wilson DC, Devlin J, Boudreau R, Robbins P, Hirsch R: Follistatin-like protein-1 is a novel proinflammatory molecule. *J Immunol* 2006, 177(7):4758-4762.

12. Wu Y, Zhou S, Smas CM: Downregulated expression of the secreted glycoprotein follistatin-like 1 (Fstl1) is a robust hallmark of preadipocyte to adipocyte conversion. *Mech Dev* 2010, 127(3-4):183-202.

13. Hao K, Niu T, Xu X, Fang Z: Single-nucleotide polymorphisms of the KCNS3 gene are significantly associated with airway hyperresponsiveness. *Hum Genet* 2005, 116(5):378-383.

14. Kinyamu HK, Collins JB, Grissom SF, Hebbar PB, Archer TK: Genome wide transcriptional profiling in breast cancer cells reveals distinct changes in hormone receptor target genes and chromatin modifying enzymes after proteasome inhibition. *Mol Carcinog* 2008, 47(11):845-885.

15. Spira A, Beane J, Pinto-Plata V, Kadar A, Liu G, Shah V, Celli B, Brody JS: Gene expression profiling of human lung tissue from smokers with severe emphysema. *Am J Respir Cell Mol Biol* 2004, 31(6):601-610.

16. Kim SH, Kim YK, Park HW, Jee YK, Kim SH, Bahn JW, Chang YS, Kim SH, Ye YM, Shin ES *et al*: Association between polymorphisms in prostanoid receptor genes and aspirin-intolerant asthma. *Pharmacogenet Genomics* 2007, 17(4):295-304.

17. Bosco A, Holt PG: Genome-wide expression profiling of T-cells in childhood wheeze. *Eur Respir J* 2008, 32(5):1138-1140.

18. Hinson ER, Joshi NS, Chen JH, Rahner C, Jung YW, Wang X, Kaech SM, Cresswell P: Viperin is highly induced in neutrophils and macrophages during acute and chronic lymphocytic choriomeningitis virus infection. *J Immunol* 2010, 184(10):5723-5731.

19. Machesky NJ, Zhang G, Raghavan B, Zimmerman P, Kelly SL, Merrill AH, Jr., Waldman WJ, Van Brocklyn JR, Trgovcich J: Human cytomegalovirus regulates bioactive sphingolipids. *J Biol Chem* 2008, 283(38):26148-26160.

20. Bird AD, Tan KH, Olsson PF, Zieba M, Flecknoe SJ, Liddicoat DR, Mollard R, Hooper SB, Cole TJ: Identification of glucocorticoid-regulated genes that control cell proliferation during murine respiratory development. *J Physiol* 2007, 585(Pt 1):187-201.
