# Supplementary material for: Vitamin D-responsive SGPP2 variants associated with lung cell expression and lung function
Source: BMC Med Genet. 2013 Nov 25;14:122. doi: 10.1186/1471-2350-14-122 (PMC3907038; doi:10.1186/1471-2350-14-122)
Supplement: Additional file 3: Table S2 — The distribution of studied SNPs in Thirteen Vitamin D-responsive Genes for European- and African-American Ancestry Groups in the Health ABC Cohort Study. [file 1471-2350-14-122-S3.docx]

**Additional file 3: Table S2.** The distribution of studied SNPs in Thirteen Vitamin D-responsive Genes for European- and African-American Ancestry Groups in the Health ABC Cohort Study

| **Gene** | **Chromosomal Position** | **EntrezGene ID** | **Size (bp)*** | **# SNPs in European-Americans**** | **# SNPs in African-Americans**** |
| --- | --- | --- | --- | --- | --- |
| ***CST6*** | 11q13 | 1474 | 7513 | 2 | 2 |
| ***DAPK1*** | 9q34.1 | 1612 | 216792 | 124 | 121 |
| ***DTX4*** | 11q12.1 | 23220 | 42200 | 11 | 10 |
| ***EMB*** | 5q11.1 | 133418 | 48724 | 11 | 11 |
| ***FSTL1*** | 3q13.33 | 11167 | 62698 | 22 | 24 |
| ***KAL1*** | Xp22.32 | 3730 | 209311 | 35 | 47 |
| ***KCNS3*** | 2p24 | 3790 | 60279 | 22 | 25 |
| ***KLF4*** | 9q31 | 9314 | 10620 | 1 | 1 |
| ***PTGER2*** | 14q22 | 5732 | 20207 | 24 | 37 |
| ***RSAD2*** | 2p25.2 | 91543 | 26567 | 9 | 11 |
| ***SGPP2*** | 2q36.1 | 130367 | 140294 | 40 | 46 |
| ***SLITRK6*** | 13q31.1 | 84189 | 12561 | 7 | 9 |
| ***TMEM40*** | 3p25.2 | 55287 | 31416 | 5 | 11 |
|  |  |  |  | Total = 313 | 355 |

*Includes 3,000 bp at 3’ and 5’ ends of gene

** SNPs filtered for Minor Allele Frequency and Hardy-Weinberg Equilibrium
