# Supplementary material for: Vitamin D-responsive SGPP2 variants associated with lung cell expression and lung function
Source: BMC Med Genet. 2013 Nov 25;14:122. doi: 10.1186/1471-2350-14-122 (PMC3907038; doi:10.1186/1471-2350-14-122)
Supplement: Additional file 4: Table S3 — SNP by 25(OH)D interactions associated with the FEV1 phenotype in a) European-Americans, and b) African-Americans. [file 1471-2350-14-122-S4.docx]

**Additional file 4: Table S3.** SNP by 25(OH)D interactions associated with the FEV_1_ phenotype in a) European-Americans, and b) African-Americans.

**a) European-Americans**

|  |  |  |  | **Predicted FEV_1_ difference (mL) by serum 25(OH)D** | | |
| --- | --- | --- | --- | --- | --- | --- |
| **Gene** | **SNP** | **Interaction Coefficient β_Interaction_**** | **Nominal P-value*** | **12 ng/ml** | **20 ng/ml** | **30 ng/ml** |
| ***DAPK1*** | rs2378753 | -6.98 | 4.00x10^-03^ | 108.6*** | 52.8 | -17.0 |
|  | rs3095747 | -7.68 | 5.17x10^-03^ | 111.2 | 49.8 | -26.9 |
| ***KAL1*** | rs5933673 | 5.77 | 9.70x10^-03^ | -121.7 | -75.5 | -17.8 |
| ***SGPP2*** | rs13021671† | -4.97 | 1.90x10^-02^ | 52.5 | 12.8 | -36.9 |
|  | rs2009150 | 5.91 | 2.42x10^-02^ | -67.1 | -19.9 | 39.2 |
|  | rs6714352† | 6.05 | 3.15x10^-02^ | -86.2 | -37.7 | 22.7 |
|  | rs735678† | -8.31 | 4.45x10^-02^ | 108.0 | 42.0 | -41.0 |
| ***SLITRK6*** | rs1337267 | -3.05 | 4.54x10^-02^ | 58.3 | 33.9 | 3.3 |
|  | rs356279 | -3.25 | 4.82x10^-02^ | 53.6 | 27.6 | -5.0 |
|  | rs631906 | -3.05 | 4.82x10^-02^ | 58.3 | 33.9 | 3.3 |

* Nominal p-values are from additive models, adjusted for age, height, smoking, gender, study site, ancestry principal components, season of vitamin D measurement, and serum 25(OH)D.

**Interaction regression coefficient compares individuals heterozygous or homozygous for the minor allele (≥1 copy of the minor allele) to individuals with the homozygous wild-type genotype (i.e., no copies of the minor allele)

***Illustrative interpretation: In participants with serum 25(OH)D of 12 ng/mL, participants ≥1 copy of the minor allele had an estimated mean FEV_1_ **108 mL higher** than homozygous wild-type individuals

† This SNP has a significant SNP by serum 25(OH)D interaction for both the FEV_1_ and FEV_1_/FVC phenotypes in European-Americans

**b) African-Americans**

|  |  |  | | **Predicted FEV_1_ difference (mL) by serum 25(OH)D** | | |
| --- | --- | --- | --- | --- | --- | --- |
| ***Gene*** | **SNP** | **β_Interaction_**** | **Nominal P-value*** | **12 ng/ml** | **20 ng/ml** | **30 ng/ml** |
| ***DAPK1*** | rs1056719 | 5.76 | 2.87x10^-02^ | -67.96*** | -21.89 | 35.70 |
|  | rs11141934 | 14.62 | 2.04x10^-02^ | -91.70 | 25.23 | 171.40 |
|  | rs3128519 | 5.97 | 2.07x10^-02^ | -61.26 | -13.49 | 46.24 |
|  | rs10868609 | -8.43 | 3.47x10^-02^ | 95.62 | 28.21 | -56.04 |
|  | rs3128477 | -6.11 | 4.04x10^-02^ | 49.62 | 0.70 | -60.44 |
|  | rs10512187 | 5.57 | 4.36x10^-02^ | -79.76 | -35.24 | 20.42 |
| ***DTX4*** | rs12284698† | 9.52 | 2.70x10^-02^ | -54.63 | 21.50 | 116.65 |
|  | rs1048444 | -5.27 | 5.43x10^-03^ | 44.21 | 2.04 | -50.67 |
|  | rs656163 | -5.67 | 8.44x10^-03^ | 66.63 | 21.30 | -35.37 |
| ***EMB*** | rs13159894 | -10.10 | 2.77x10^-02^ | 97.08 | 16.25 | -84.79 |
|  | rs16879113 | -10.10 | 2.77x10^-02^ | 97.08 | 16.25 | -84.79 |
|  | rs7729211† | 11.74 | 2.82x10^-03^ | -84.71 | 9.24 | 126.69 |
| ***FSTL1*** | rs2673704 | 10.71 | 3.75x10^-02^ | -21.76 | 63.90 | 170.97 |
| ***KAL1*** | rs7051071† | -8.50 | 3.17x10^-02^ | 105.89 | 37.88 | -47.14 |
|  | rs10127300† | -5.04 | 3.24x10^-02^ | 43.06 | 2.75 | -47.63 |
|  | rs5978935† | -5.03 | 3.30x10^-02^ | 41.42 | 1.16 | -49.16 |
|  | rs5933677 | 7.53 | 6.61x10^-03^ | -56.64 | 3.61 | 78.92 |
|  | rs5978934 | 5.88 | 7.81x10^-03^ | -61.70 | -14.65 | 44.16 |
|  | rs5933668 | 8.37 | 3.06x10^-03^ | -122.50 | -55.57 | 28.08 |
|  | rs6530187† | 6.79 | 7.15x10^-03^ | -66.38 | -12.02 | 55.93 |
|  | rs5978943 | -8.07 | 8.10x10^-03^ | 83.65 | 19.07 | -61.66 |
| ***KCNS3*** | rs1461949 | -7.77 | 4.91x10^-02^ | 69.93 | 7.79 | -69.89 |
|  | rs1870822 | -8.17 | 2.16x10^-02^ | 35.66 | -29.69 | -111.37 |
|  | rs4832524 | -8.10 | 4.11x10^-02^ | 75.68 | 10.91 | -70.04 |
|  | rs7583266 | -8.53 | 5.18x10^-03^ | 51.12 | -17.12 | -102.41 |
| ***PTGER2*** | rs10136396 | -8.87 | 2.89x10^-02^ | 91.80 | 20.88 | -67.78 |
|  | rs10136414 | -8.94 | 2.82x10^-02^ | 91.94 | 20.41 | -69.01 |
|  | rs10151916 | -8.91 | 2.83x10^-02^ | 89.87 | 18.61 | -70.46 |
|  | rs11851457 | -8.42 | 3.66x10^-02^ | 91.94 | 24.58 | -59.63 |
|  | rs12587363 | -8.68 | 4.88x10^-02^ | 86.47 | 17.06 | -69.71 |
|  | rs12590616 | -8.18 | 2.84x10^-02^ | 79.80 | 14.32 | -67.53 |
|  | rs1254598† | 8.11 | 1.30x10^-02^ | -78.28 | -13.41 | 67.68 |
|  | rs708499 | -10.02 | 1.53x10^-02^ | 106.51 | 26.33 | -73.89 |
|  | rs708498 | -9.30 | 2.19x10^-02^ | 109.09 | 34.66 | -58.37 |
|  | rs10142849 | -9.11 | 2.51x10^-02^ | 96.62 | 23.75 | -67.33 |
|  | rs28613641 | -8.26 | 4.14x10^-02^ | 84.68 | 18.61 | -63.98 |
|  | rs12587410 | -8.87 | 4.25x10^-02^ | 91.80 | 20.88 | -67.78 |
| ***SGPP2*** | rs7559017 | 6.40 | 4.13x10^-02^ | -29.40 | 21.82 | 85.85 |
|  | rs10176933 | -8.41 | 4.52x10^-02^ | 77.59 | 10.35 | -73.70 |
|  | rs4674662 | 6.03 | 5.00x10^-02^ | -23.96 | 24.29 | 84.60 |
|  | rs4673024† | 9.02 | 1.40x10^-03^ | -45.95 | 26.22 | 116.42 |
|  | rs1436786 | 7.84 | 2.40x10^-04^ | -28.37 | 34.34 | 112.74 |
| ***SLITRK6*** | rs431057 | 10.52 | 3.28x10^-02^ | -76.38 | 7.77 | 112.95 |

* Nominal p-values are from additive models, adjusted for age, height, smoking, gender, study site, ancestry principal components, season of vitamin D measurement, and serum 25(OH)D.

**Interaction regression coefficient compares individuals heterozygous or homozygous for the minor allele (≥1 copy of the minor allele) to individuals with the homozygous wild-type genotype (i.e., no copies of the minor allele)

***Illustrative interpretation: In participants with serum 25(OH)D of 12 ng/mL, participants ≥1 copy of the minor allele had an estimated mean FEV_1_ **68 mL lower** than homozygous wild-type individuals

† This SNP has a significant SNP by serum 25(OH)D interaction for both the FEV_1_ and FEV_1_/FVC phenotypes in African-Americans
