# Supplementary material for: Vitamin D-responsive SGPP2 variants associated with lung cell expression and lung function
Source: BMC Med Genet. 2013 Nov 25;14:122. doi: 10.1186/1471-2350-14-122 (PMC3907038; doi:10.1186/1471-2350-14-122)
Supplement: Additional file 5: Table S4 — SNP by serum 25(OH)D interactions in association with the FEV1/FVC phenotype in a) European-Americans, and b) African-Americans. [file 1471-2350-14-122-S5.docx]

**Additional file 5: Table S4.** SNP by serum 25(OH)D interactions in association with the FEV_1_/FVC phenotype in a) European-Americans, and b) African-Americans.

**a) European-Americans**

|  |  |  | | **Predicted FEV_1_/FVC difference by serum 25(OH)D** | | |
| --- | --- | --- | --- | --- | --- | --- |
| **Gene** | **SNP** | **β_Interaction_**** | **Nominal P-value*** | **12 ng/mL** | **20 ng/mL** | **30 ng/mL** |
| ***KAL1*** | rs1079854 | 0.074 | 4.59X10^-02^ | -1.86*** | -1.27 | -0.53 |
|  | rs11095490 | 0.078 | 3.37X10^-02^ | -1.88 | -1.26 | -0.48 |
|  | rs12840575 | 0.074 | 4.59X10^-02^ | -1.86 | -1.27 | -0.53 |
|  | rs1859867 | 0.068 | 3.84X10^-02^ | 1.45 | 0.84 | 2.72 |
| ***PTGER2*** | rs2229187 | 0.164 | 3.87X10^-02^ | -2.62 | -1.31 | 0.33 |
| ***SGPP2*** | rs13021671 | -0.090 | 1.62X10^-02^ | 1.29 | 0.57 | -0.33 |
|  | rs4416206 | 0.088 | 3.04X10^-02^ | -1.48 | -0.78 | 0.10 |
|  | rs6714352 | 0.105 | 2.12X10^-02^ | -1.19 | -0.35 | 0.70 |
|  | rs6758392 | 0.085 | 2.98X10^-02^ | -1.31 | -0.63 | 0.23 |
|  | rs735678 | -0.118 | 5.10X10^-03^ | 1.94 | 0.99 | -0.19 |
| ***TMEM40*** | rs9876483 | -0.122 | 1.58X10^-02^ | 2.21 | 1.24 | 0.02 |

* Nominal p-values are from additive models, adjusted for age, height, smoking, gender, study site, ancestry principal components, season of vitamin D measurement, and serum 25(OH)D.

**Interaction regression coefficient compares individuals heterozygous or homozygous for the minor allele (≥1 copy of the minor allele) to individuals with the homozygous wild-type genotype (i.e., no copies of the minor allele)

***Illustrative interpretation: In participants with serum 25(OH)D of 12 ng/mL, participants ≥1 copy of the minor allele had an estimated mean FEV_1_/FVC ratio **1.86 lower** than homozygous wild-type individuals

1. **African-Americans**

|  |  |  | | **Predicted FEV_1_/FVC difference by serum 25(OH)D** | | |
| --- | --- | --- | --- | --- | --- | --- |
| **Gene** | **SNP** | **β_Interaction_**** | **Nominal P-value*** | **12 ng/mL** | **20 ng/ml** | **30 ng/mL** |
| ***DAPK1*** | rs3118867 | -0.088 | 2.68X10^-02^ | 0.22*** | -0.48 | -1.36 |
|  | rs3818584 | -0.135 | 3.79X10^-02^ | 0.55 | -0.52 | -1.87 |
|  | rs4878115 | -0.133 | 2.03X10^-02^ | 0.58 | -0.48 | -1.82 |
|  | rs1927975 | -0.128 | 3.17X10^-02^ | 0.25 | -0.78 | -2.06 |
|  | rs2274605 | -0.138 | 3.48X10^-02^ | 0.62 | -0.48 | -1.86 |
|  | rs943855 | -0.131 | 4.10X10^-02^ | 0.50 | -0.55 | -1.85 |
| ***DTX4*** | rs12284698 | 0.159 | 3.77X10^-02^ | -0.04 | 1.23 | 2.82 |
| ***EMB*** | rs7729211 | 0.173 | 1.33X10^-02^ | -1.91 | -0.52 | 1.21 |
| ***FSTL1*** | rs1105220 | -0.148 | 2.68X10^-02^ | 1.78 | 0.60 | -0.88 |
|  | rs1624195 | -0.117 | 3.45X10^-02^ | 1.24 | 0.31 | -0.86 |
|  | rs4533682 | -0.143 | 3.57X10^-02^ | 1.53 | 0.39 | -1.04 |
| ***KAL1*** | rs6530187 | 0.155 | 3.68X10^-02^ | -1.07 | 0.17 | 1.72 |
|  | rs5978934 | -0.144 | 2.22X10^-02^ | 0.97 | -0.18 | -1.62 |
|  | rs6640194 | -0.104 | 2.52X10^-02^ | -0.01 | -0.84 | -1.88 |
|  | rs5978943 | 0.143 | 3.11X10^-02^ | -0.85 | 0.29 | 1.73 |
|  | rs10127300 | -0.078 | 4.04X10^-02^ | 0.96 | 0.34 | -0.43 |
|  | rs5978935 | -0.077 | 4.11X10^-02^ | 0.93 | 0.31 | -0.46 |
|  | rs7887099 | -0.094 | 4.18X10^-02^ | 0.08 | -0.67 | -1.61 |
|  | rs7051071 | -0.184 | 4.42X10^-02^ | 0.77 | -0.70 | -2.54 |
| ***KCNS3*** | rs3747516 | 0.145 | 2.02X10^-02^ | -1.48 | -0.32 | 1.13 |
| ***PTGER2*** | rs1254581 | -0.150 | 2.90X10^-02^ | 0.31 | -0.89 | -2.39 |
|  | rs1495785 | -0.098 | 4.99X10^-02^ | 0.08 | -0.70 | -1.68 |
|  | rs1254598 | 0.121 | 2.42X10^-02^ | -1.12 | -0.15 | 1.06 |
| ***SGPP2*** | rs17562982 | -0.118 | 3.68X10^-02^ | 0.41 | -0.54 | -1.72 |
|  | rs2009150 | 0.164 | 1.18X10^-02^ | -1.95 | -0.65 | 0.99 |
|  | rs4673024 | 0.181 | 1.41X10^-03^ | -1.20 | 0.25 | 2.05 |

* Nominal p-values are from additive models, adjusted for age, height, smoking, gender, study site, ancestry principal components, season of vitamin D measurement, serum 25(OH)D.

**Interaction regression coefficient compares individuals heterozygous or homozygous for the minor allele (≥1 copy of the minor allele) to individuals with the homozygous wild-type genotype (i.e., no copies of the minor allele)

***Illustrative interpretation: In participants with serum 25(OH)D of 12 ng/mL, participants ≥1 copy of the minor allele had an estimated mean FEV_1_ **0.22 higher** than homozygous wild-type individuals
