# Supplementary figures and images for: Vitamin D-responsive SGPP2 variants associated with lung cell expression and lung function
Source: BMC Med Genet. 2013 Nov 25;14:122. doi: 10.1186/1471-2350-14-122 (PMC3907038; doi:10.1186/1471-2350-14-122)

**Additional file 6: Figure S1.** Genome-wide Quantile-Quantile Plot for *SGPP2* eQTL findings


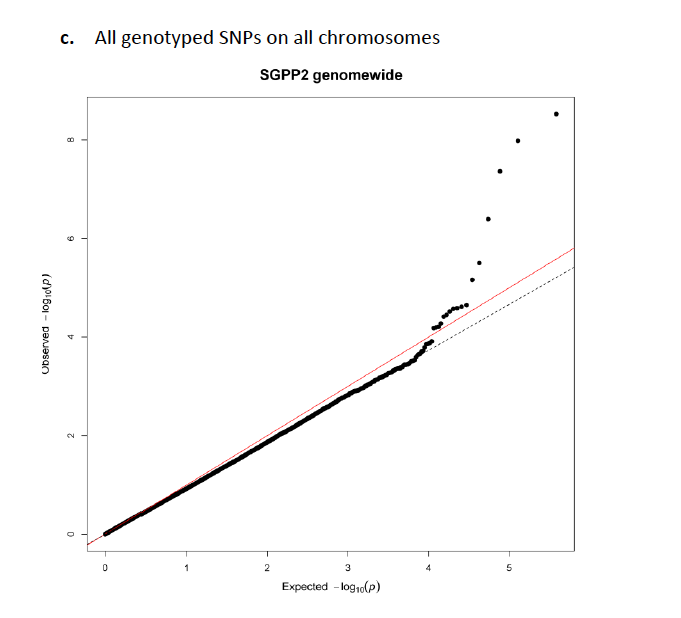

Supplement: Additional file 6: Figure S1 — Genome-wide Quantile-Quantile Plot for SGPP2 eQTL findings. [file 1471-2350-14-122-S6.docx]

**Additional file 7: Figure S2.** Genome-wide Manhattan Plot for *SGPP2* eQTL findings


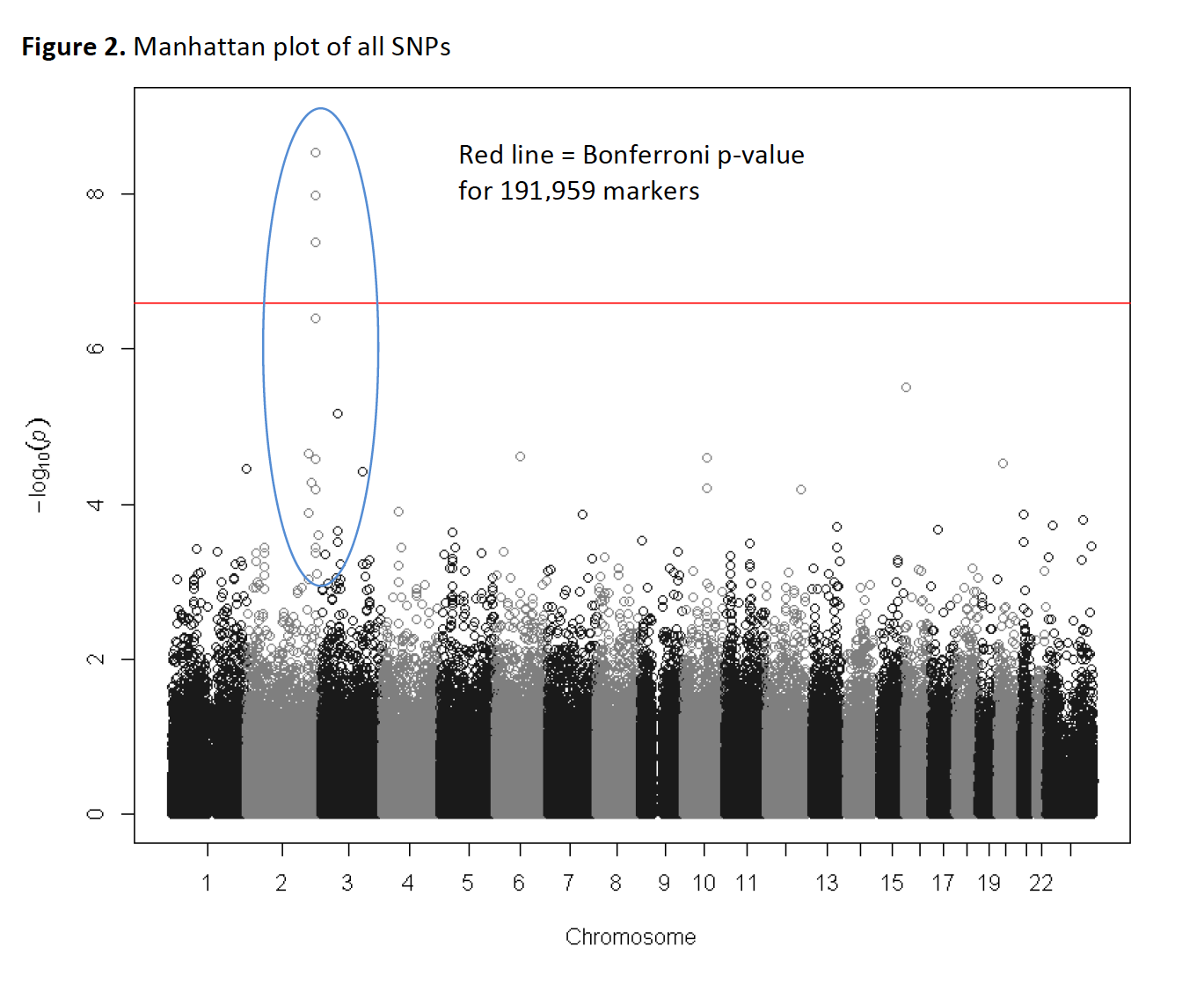

Supplement: Additional file 7: Figure S2 — Genome-wide Manhattan Plot for SGPP2 eQTL findings. [file 1471-2350-14-122-S7.docx]
