# Supplementary material for: Vitamin D-responsive SGPP2 variants associated with lung cell expression and lung function
Source: BMC Med Genet. 2013 Nov 25;14:122. doi: 10.1186/1471-2350-14-122 (PMC3907038; doi:10.1186/1471-2350-14-122)
Supplement: Additional file 8: Table S5 — The most statistically significant associations (nominal P < 2.0 × 10^-02^) between single nucleotide polymorphisms in vitamin D-responsive genes and FEV1 for a) European-Americans and b) African-Americans (all SNPs, including redundant SNPs are shown). [file 1471-2350-14-122-S8.docx]

**Additional file 8: Table S5.** The most statistically significant associations (nominal P<2.0x10^-02^) between single nucleotide polymorphisms in vitamin D-responsive genes and FEV_1_ for a) European-Americans and b) African-Americans (all SNPs, including redundant SNPs are shown).

1. **European-Americans**

| **Gene** | **RS#** | **Chr** | **Coded Allele** | **Coded Allele Frequency (%)** | **Beta (mL)** | **Standard Error (mL)** | **Nominal P** | **Model** |
| --- | --- | --- | --- | --- | --- | --- | --- | --- |
| ***DAPK1*** | rs11141878 | 9 | A | 36 | -103.98 | 36.33 | 4.26x10^-03^ | R |
|  | rs4877361 | 9 | G | 14 | 72.47 | 27.36 | 8.17x10^-03^ | D |
|  | rs17477827 | 9 | A | 14 | 70.66 | 27.33 | 9.82x10^-03^ | D |
|  | rs4878089 | 9 | A | 46 | 39.68 | 16.93 | 1.92x10^-02^ | A |
| ***SGPP2*** | rs4674656 | 2 | A | 25 | -58.70 | 19.67 | 2.88x10^-03^ | A |

1. **African-Americans**

| **Gene** | **RS#** | **Chr** | **Coded Allele** | **Coded Allele Frequency (%)** | **Beta (mL)** | **Standard Error (mL)** | **Nominal P** | **Model** |
| --- | --- | --- | --- | --- | --- | --- | --- | --- |
| ***DAPK1*** | rs3128491 | 9 | G | 33 | 51.48 | 21.44 | 1.65X10^-02^ | A |
| ***FSTL1*** | rs4676781† | 3 | T | 8 | -110.13 | 35.34 | 1.88X10^-03^ | A |
|  | rs13100865† | 3 | G | 9 | -105.96 | 35.02 | 2.54X10^-03^ | A |
|  | rs13097755† | 3 | T | 28 | -60.46 | 21.59 | 5.20X10^-03^ | A |
|  | rs2272515† | 3 | C | 28 | -60.46 | 21.59 | 5.20X10^-03^ | A |
| ***KAL1*** | rs6530200 | 23 | T | 47 | -45.28 | 16.81 | 7.20X10^-03^ | A |
|  | rs974655 | 23 | A | 49 | 79.22 | 30.33 | 9.14X10^-03^ | D |
| ***KCNS3*** | rs3747515 | 2 | T | 16 | 243.92 | 83.47 | 3.56X10^-03^ | R |
|  | rs1031771 | 2 | A | 16 | 243.76 | 83.52 | 3.60X10^-03^ | R |
| ***RSAD2*** | rs4669114 | 2 | G | 10 | -119.55 | 36.20 | 9.93X10^-04^ | D |
|  | rs10495546 | 2 | C | 10 | -119.08 | 36.18 | 1.03X10^-03^ | D |
|  | rs4669113 | 2 | C | 10 | -119.08 | 36.18 | 1.03X10^-03^ | D |
|  | rs6431837 | 2 | C | 47 | -101.06 | 33.55 | 2.66X10^-03^ | R |
|  | rs7570384 | 2 | C | 38 | -55.35 | 20.05 | 5.88X10^-03^ | A |
|  | rs4669111 | 2 | A | 41 | -49.75 | 20.07 | 1.34X10^-02^ | A |
| ***SGPP2*** | rs4528748† | 2 | C | 27 | -209.95 | 54.10 | 1.11X10^-04^* | R |
|  | rs7556867† | 2 | G | 27 | -207.89 | 54.48 | 1.44X10^-04^* | R |
|  | rs6758392† | 2 | T | 28 | -182.36 | 51.94 | 4.67X10^-04^* | R |

* = FDR q-value <5.0x10^-02^

Model adjusted for age, height, smoking, gender, study site, and ancestry principal components.

A=additive model, D=Dominant model, R=recessive model

† SNP is nominally significant (P<2.0x10^-02^) for both FEV_1_ and FEV_1_/FVC phenotypes in African-Americans
