# Supplementary material for: Vitamin D-responsive SGPP2 variants associated with lung cell expression and lung function
Source: BMC Med Genet. 2013 Nov 25;14:122. doi: 10.1186/1471-2350-14-122 (PMC3907038; doi:10.1186/1471-2350-14-122)
Supplement: Additional file 9: Table S6 — The most statistically significant associations (nominal P < 2.0 × 10^-02^) between single nucleotide polymorphisms in vitamin D-responsive genes and the FEV1/FVC ratio for a) European-Americans and b) African-Americans in the Health ABC cohort. [file 1471-2350-14-122-S9.docx]

**Additional file 9: Table S6.** The most statistically significant associations (nominal P<2.0x10^-02^) between single nucleotide polymorphisms in vitamin D-responsive genes and the FEV_1_/FVC ratio for a) European-Americans and b) African-Americans in the Health ABC cohort

**a) European-Americans**

| **Gene** | **RS#** | **Chr** | **Coded Allele** | **Coded Allele Frequency** | **Beta (%)** | **Standard Error (%)** | **Nominal P** | **Model** |
| --- | --- | --- | --- | --- | --- | --- | --- | --- |
| ***KLF4*** | rs2236599 | 9 | A | 19 | -0.85 | 0.33 | 1.15x10^-02^ | A |

**b) African-Americans**

| **Gene** | **RS#** | **Chr** | **Coded Allele** | **Coded Allele Frequency (%)** | **Beta (%)** | **Standard Error (%)** | **Nominal P** | **Model** |
| --- | --- | --- | --- | --- | --- | --- | --- | --- |
| ***FSTL1*** | rs4676781 | 3 | T | 8 | -1.92 | 0.67 | 4.47x10^-03^ | A |
|  | rs13100865 | 3 | G | 9 | -1.81 | 0.67 | 6.65x10^-03^ | A |
|  | rs13097755 | 3 | T | 28 | -1.03 | 0.41 | 1.27x10^-02^ | A |
|  | rs2272515 | 3 | C | 28 | -1.03 | 0.41 | 1.27x10^-02^ | A |
| ***KAL1*** | rs5933668 | 23 | T | 19 | -2.76 | 0.86 | 1.32x10^-03^ | R |
|  | rs1859867 | 23 | C | 40 | 0.95 | 0.32 | 3.13x10^-03^ | A |
|  | rs1079854 | 23 | G | 38 | 1.83 | 0.63 | 3.65x10^-03^ | R |
|  | rs2108400 | 23 | C | 38 | 1.81 | 0.63 | 4.11x10^-03^ | R |
|  | rs4830593 | 23 | A | 38 | 1.80 | 0.63 | 4.22x10^-03^ | R |
|  | rs16998683 | 23 | C | 12 | 1.82 | 0.70 | 9.75x10^-03^ | D |
| ***SGPP2*** | rs4597517 | 2 | A | 23 | -3.30 | 1.21 | 6.71x10^-03^ | R |
|  | rs4528748 | 2 | C | 27 | -1.15 | 0.43 | 7.70x10^-03^ | A |
|  | rs7556867 | 2 | G | 27 | -1.15 | 0.43 | 8.18x10^-03^ | A |
|  | rs6758392 | 2 | T | 28 | -1.10 | 0.43 | 1.01x10^-02^ | A |

Model adjusted for age, height, smoking, gender, study site, and ancestry principal components.

A=additive model, D=Dominant model, R=recessive model
