# Supplementary material for: Vitamin D-responsive SGPP2 variants associated with lung cell expression and lung function
Source: BMC Med Genet. 2013 Nov 25;14:122. doi: 10.1186/1471-2350-14-122 (PMC3907038; doi:10.1186/1471-2350-14-122)
Supplement: Additional file 10: Table S7 — Gene-level replication of Health ABC European-American SNP associations with FEV1 using the Framingham Heart Study cohort. [file 1471-2350-14-122-S10.docx]

**Additional file 10: Table S7.** Gene-level replication of Health ABC European-American SNP associations with FEV_1_ using the Framingham Heart Study cohort*

| **FEV_1_ Phenotype** | | | | | | |  |
| --- | --- | --- | --- | --- | --- | --- | --- |
| **Gene** | **Total # of FHS SNPs** | **# SNPs with p<0.05** | **Most Significant SNP in Gene** | | | | |
|  |  |  | **RS#** | **MAF (%)** | **Beta (mL)** | **Nominal P** | |
| *SGPP2* | 145 | 23 | rs10932956 | 21 | 29.2 | 2.23 x10^-02^ | |
| *DAPK1* | 340 | 23 | rs7025760 | 21 | 23.6 | 1.86x10^-02^ | |

*all models use additive genetic coding.
