# Supplementary material for: Vitamin D-responsive SGPP2 variants associated with lung cell expression and lung function
Source: BMC Med Genet. 2013 Nov 25;14:122. doi: 10.1186/1471-2350-14-122 (PMC3907038; doi:10.1186/1471-2350-14-122)
Supplement: Additional file 11: Table S8 — Gene Ontology of Thirteen Nominally Significant Candidate Genes from the UniProtKb-GOA Database (http://www.ebi.ac.uk/QuickGO/). [file 1471-2350-14-122-S11.docx]

**Additional file 11: Table S8.** Gene Ontology of Thirteen Nominally Significant Candidate Genes from the UniProtKb-GOA Database (http://www.ebi.ac.uk/QuickGO/)

| Gene | Gene Name | Function(s) | Pathway(s) | Location(s) |
| --- | --- | --- | --- | --- |
| *CST6* | Cystatin E/M | cysteine-type endopeptidase inhibitor | anatomical structure morphogenesis | cornified envelope, extracellular region |
| *DAPK1* | Death-associated protein kinase 1 | ATP and calmodulin binding | intracellular protein kinase cascade, apoptosis regulation | actin cytoskeleton |
| *DTX4* | Deltex homolog 4 | zinc ion binding | Notch signaling pathway | cytoplasm |
| *EMB* | Embigin | N/A | cell adhesion | integral membrane protein |
| *FSTL1* | Follistatin-like 1 | calcium ion binding, heparin binding | Bone morphogenetic protein signaling pathway | Extracellular space |
| *KAL1* | Kallmann syndrome 1 sequence | extracellular matrix structural component, serine-type endopeptidase inhibitor | axon guidance, chemotaxis, cell movement, cell adhesion | cell surface, extracellular space |
| *KCNS3* | Potassium voltage-gated channel, delayed-rectifier, subfamily S, member 3 | delayed-rectifier potassium channel | potassium ion transport, regulation of insulin secretion | Golgi and plasma membrane |
| *KLF4* | Kruppel-like factor 4 | transcription repressor activity | regulation of cell proliferation, mesodermal cell fate determination | nuclear |
| *PTGER2* | Prostaglandin E receptor 2 (subtype EP2) | G protein coupled receptor for prostaglandin E | GPCR signaling, regulation of cell proliferation | integral to plasma membrane |
| *RSAD2* | Radical S-adenosyl methionine domain containing 2 | iron-sulfur cluster binding, metal ion binding | defense response to virus | endoplasmic reticulum |
| *SGPP2* | Sphingosine-1-phosphate phosphatase 2 | sphingosine-1-phosphate phosphatase activity | sphingosine metabolic process | endoplasmic reticulum membrane |
| *SLITRK6* | SLIT and NTRK-like family, member 6 | N/A | axonogenesis | integral membrane protein |
| *TMEM40* | Transmembrane protein 40 | N/A | N/A | integral membrane protein |
