# Supplementary material for: Vitamin D-responsive SGPP2 variants associated with lung cell expression and lung function
Source: BMC Med Genet. 2013 Nov 25;14:122. doi: 10.1186/1471-2350-14-122 (PMC3907038; doi:10.1186/1471-2350-14-122)
Supplement: Additional file 12: Table S9 — Evidence Supporting the Role in Lung Health and/or Regulation by Glucocorticoids For Genes Differentially Expressed by Serum Vitamin D. [file 1471-2350-14-122-S12.docx]

**Additional file 12: Table S9.** Evidence* Supporting the Role in Lung Health and/or Regulation by Glucocorticoids For Genes Differentially Expressed by Serum Vitamin D

| Gene Name: | **Asthma** | **COPD** | **Inflammation** | **Infection Defense** | **Regulation by Glucocorticoids** |
| --- | --- | --- | --- | --- | --- |
| *EMB* |  |  |  | **[**[**10**](#_ENREF_10)**]*** | **[**[**10**](#_ENREF_10)**]** |
| *FSTL1* |  |  | **[**[**11**](#_ENREF_11)**]** |  | **[**[**12**](#_ENREF_12)**]** |
| *KCNS3* | **[**[**13**](#_ENREF_13)**]** |  |  |  | **[**[**14**](#_ENREF_14)**]** |
| *KLF4* |  | **[**[**15**](#_ENREF_15)**]** |  |  |  |
| *PTGER2* | **[**[**16**](#_ENREF_16)**,** [**17**](#_ENREF_17)**]** |  |  |  |  |
| *RSAD2* |  |  |  | **[**[**10**](#_ENREF_10)**,** [**18**](#_ENREF_18)**]** |  |
| *SGPP2* |  |  |  | **[**[**19**](#_ENREF_19)**]** |  |
| *SLITRK6* |  |  |  |  | **[**[**20**](#_ENREF_20)**]** |

***** Numbers correspond to bibliographic citations**.**
